# Modulation of Antibody Responses to the V1V2 and V3 Regions of HIV-1 Envelope by Immune Complex Vaccines

**DOI:** 10.3389/fimmu.2018.02441

**Published:** 2018-10-26

**Authors:** Catarina E. Hioe, Rajnish Kumar, Chitra Upadhyay, Muzafar Jan, Alisa Fox, Vincenza Itri, Kristina K. Peachman, Mangala Rao, Lily Liu, Nathan C. Lo, Michael Tuen, Xunqing Jiang, Xiang-Peng Kong, Susan Zolla-Pazner

**Affiliations:** ^1^Icahn School of Medicine at Mount Sinai, New York, NY, United States; ^2^James J. Peters VA Medical Center, Bronx, NY, United States; ^3^United States Military HIV Research Program, Walter Reed Army Institute of Research, Silver Spring, MD, United States; ^4^Henry M. Jackson Foundation for the Advancement of Military Medicine, Bethesda, MD, United States; ^5^Department of Pathology, New York University School of Medicine, New York, NY, United States; ^6^Department of Biochemistry and Molecular Pharmacology, New York University School of Medicine, New York, NY, United States

**Keywords:** vaccine, HIV, envelope, antibody, immune complex

## Abstract

Prophylactic HIV vaccines must elicit antibodies (Abs) against the virus envelope glycoproteins (Env) to effectively prevent HIV infection. We investigated a vaccine platform that utilizes immune complexes made of Env proteins gp120 and monoclonal Abs (mAbs) against different gp120 epitopes. We previously observed alterations in V3 antigenicity upon formation of certain gp120/mAb complexes and demonstrated the ability of these complexes to modulate the elicitation of V3 Ab responses. However, the effects on the V1V2 domain, an important target for Abs that correlate with vaccine-induced protection against HIV, have not been studied, nor have immune complex vaccines made with non-B subtype Env. This study compared subtypes B (JRFL) and CRF_01.AE (A244) Env gp120 proteins in complex with selected gp120-specific mAbs. Allosteric and antigenic changes were detected on these immune complexes, indicating that gp120/mAb interaction induces alterations on the Env surface that may modify the Env immunogenic properties. To evaluate this idea, mice were immunized with gp120/mAb complexes or their uncomplexed gp120 counterparts. The overall serum IgG titers elicited against gp120 were comparable, but a marked skewing toward V1V2 or V3 was evident and dependent on the gp120 strain and the specificity of the mAb used to form the complexes. Compared with uncomplexed gp120_JRFL_, gp120_JRFL_ complexed with CD4bs or V1V2 mAbs, but not with C2 or V3 mAbs, elicited V3 Abs of greater titers and breadth, and Abs more capable of neutralizing tier 1 virus. Epitope mapping revealed a shift to a more conserved site in the V3 crown. However, the complexes did not enhance V1V2 Ab response, and the elicited V1V2 Abs were not cross-reactive. This profile contrasts with Ab responses to gp120_A244_/mAb complexes. Notably, gp120_A244_/mAb complexes induced higher levels of V1V2 Abs with some cross-reactivity, while also stimulating weak or strain-specific V3 Abs. Sera from gp120_A244_/mAb complex-immunized animals displayed no measurable virus neutralization but did mediate Ab-dependent cellular phagocytosis, albeit at levels similar to that induced by gp120_A244_ alone. These data indicate the potential utility of immune complexes as vaccines to shape Ab responses toward or away from Env sites of interest.

## Introduction

The development of HIV vaccines, which are much needed to control the HIV/AIDS pandemic, has faced tremendous scientific challenges. In 2009, the first glimmer of success was observed in the Phase III RV144 trial of the ALVAC-HIV+AIDSVAX B/E Env gp120 protein vaccine, which demonstrated 60.5% efficacy in reducing HIV acquisition at 1 year and 31.2% after 3.5 years (1, 2). The results of this trial suggest the possibility of protective effects of anti-gp120 antibody (Ab) responses. More specifically, data from an RV144 case-control study identified the presence of high levels of anti-V1V2 IgG responses as a correlate of reduced risk of HIV acquisition (3–7). Abs to V3 and Ab-dependent cellular cytotoxicity (ADCC) also inversely correlated with infection risk, albeit only in the subset of RV144 vaccine recipients who had lower levels of neutralizing Abs and Env-specific plasma IgA (4, 8, 9). Indeed, virus neutralizing activity did not correlate with reduced acquisition risk, and no tier 2 virus neutralization was measurable (3, 9). The importance of Env-specific Abs targeting V1V2 was recapitulated in studies of vaccinated macaques that were protected against challenge with neutralization-resistant SIV_MAC251_ (10–12). Nonetheless, in the RV144 trial, high levels of V1V2-specific Abs were elicited only in a fraction of vaccine recipients (3). Medium or low levels of these Abs did not correlate with lower rates of HIV acquisition, and Ab levels in recipients who demonstrated high levels of V1V2 Abs waned quickly after the final boost (3, 6, 9). A late boost 6–8 years after the initial vaccination greatly elevated anti-V1V2 Ab levels, albeit transiently (13). In the earlier VAX003 and VAX004 vaccine trials, which tested solely AIDSVAX Env gp120 proteins and showed no protective efficacy (14, 15), vaccine recipients also generated serum Abs to V1V2 and V3, but these responses peaked after 3 to 4 immunizations and declined after 5 to 7 immunizations (16). Functional Ab responses measured by tier 1 virus neutralization and ADCC similarly were not sustained. Hence, the induction of V1V2- and V3-specific Ab responses by vaccines tested in the RV144, VAX003, and VAX004 trials was not optimal, and strategies to improve the immunogenic potential of Env vaccines are warranted.

Our past studies demonstrated the capacity of certain anti-gp120 monoclonal Abs (mAbs), when administered together with gp120 proteins as immune-complex vaccines, to modulate the induction of Ab responses to V3. Specifically, immunization of mice with gp120 in the presence of anti-CD4 binding site (CD4bs) mAb 654 induced higher levels of V3-specific Abs than did immunization with gp120 alone (17–20). Enhancement of anti-V3 Ab responses was also observed following immunization with gp120 in complex with a V2 mAb, but not with a C2 mAb (20). Unlike the traditional Fc-mediated enhancement of Ab responses to immune complexes (21–23), it is the Fab-mediated activities of mAbs that induce conformational alterations in Env, as demonstrated by better V3 exposure and Ab recognition and greater proteolytic resistance, leading to increased immunogenicity (20, 24, 25). These structural changes are possible due to the uniquely dynamic nature of the gp120 structure, with flexible loops and mobile elements in its inner and outer domains, which become more stable upon interaction with its ligand CD4 or certain anti-gp120 mAbs (26–28). However, in these past studies, only immune-complex vaccines of subtype B gp120 proteins of JRFL or LAI were evaluated (17–20). Moreover, assessments of Ab responses were restricted to V3, and alterations to Ab responses against other Env regions, especially V1V2, were not examined. Nonetheless, the results of those studies indicate the potential utility of selected Env/mAb complexes to influence the elicitation of Ab responses to Env, not only increasing Ab titers but also skewing Ab specificity toward various Env regions of interest.

Studies from passive transfers of mAbs against other pathogens, such as FrCasE murine retrovirus and *Nippostrongylus bransiliensis*, in mice also provide evidence for the capacity of mAbs to modulate induction of Ab responses in terms of duration, specificity, Ig isotype, and function (29–31). In human clinical trials, administration of a mAb (Guy's 13) against adhesion protein P1 of the cariogenic dental pathogen *Streptococcus mutans* conferred long-term protection, beyond the lifetime of the transferred mAb, against *S. mutans* colonization (32). Further experiments in mice demonstrated the immunomodulatory property of mAb Guy's 13 and two other mAbs: the presence of mAb during *S. mutans* immunization elicited higher levels of endogenous Abs against protective but cryptic epitopes that inhibited bacterial adherence (33–36). This activity was mediated by the Fab fragment of the mAb, which, upon binding to P1, induced structural alterations and increased exposure of the protective cryptic epitopes, reminiscent of the enhanced Ab recognition of V3 epitopes observed in our study with anti-gp120 mAbs (17, 18, 20).

The present study was designed to further investigate how the formation of Env/mAb complexes affects the exposure or occlusion of various epitopes due to allosteric changes or sequestration of Env epitopes and to test the idea that the use of an immune complex composed of a particular pair of Env-specific mAb and Env protein as a vaccine would promote the elicitation of Ab responses that are directed toward or away from V3 and V1V2. To this end, we evaluated the antigenicity and immunogenicity of Env proteins from subtype B (gp120 B.JRFL) and CRF01_AE (gp120 AE.A244) in complex with selected mAbs specific for distinct gp120 sites, including the second constant region (C2), the V1V2 domain near the integrin α4β7 binding motif (V2i), the CD4 binding site (CD4bs), or the V3 crown (V3). Of note, gp120 AE.A244 was one of the two AIDSVAX gp120 proteins used in the RV144 and VAX003 trials (1, 15). The complexes were first examined for antigenic changes relative to the uncomplexed gp120; thereby, immune complexes made of gp120 B.JRFL and gp120 AE.A244 were probed *in vitro* for reactivity with a panel of anti-gp120 mAbs, to detect allosteric and antigenic alterations triggered on the gp120 surface upon immune complex formation. Subsequently, mice were immunized with each of the complexes vs. gp120 alone. An immune complex made of a non-native trimeric Env gp140 of subtype C (C.CN54) was also compared with its uncomplexed counterpart in another set of immunization experiment. Sera were analyzed for binding IgG to gp120, V3, and V1V2 in direct and competitive ELISAs. To detect shifts in Ab responses to sites within V3 and V1V2, epitope mapping was performed with overlapping peptides. Immune sera were also compared for their antiviral potential, including neutralization against a tier 1 virus sensitive to V3 Abs and V1V2 Abs, α4β7-Env blocking activity, and Ab-dependent cellular phagocytosis (ADCP). The data provide evidence for the use of selected anti-gp120 mAbs as valuable tools to modify the immunogenicity of Env protein vaccines, resulting in enhanced or reduced elicitation of Ab responses to V1V2 or V3.

## Materials and methods

### Antigens and mAbs

Recombinant Env proteins were obtained from the following sources: Vaccine Research and Development Branch of Division of AIDS, NIAID, NIH, USA (gp120 B.JRFL); Global Solutions for Infectious Diseases and Dr. Barton Haynes, Duke University (gp120 AE.A244); Polymum Scientific (gp140 C.CN54). MAbs for constructing immune complexes and for probing immune complex antigenicity were all human IgG1, but differed in their antigenic specificities. MAbs were purified by protein A or G columns. Peptides were obtained from Dr. Nico Karasavvas (Armed Forces Research Institute of Medical Sciences, Thailand) or custom-made by Sigma. V1V2-tags of C.1086, A.Q23, and AE.244 were gifts of Drs. Barton Haynes, Larry Liao, and Kevin Saunders (Duke University). MAb CH01 was given by Dr. Barton Haynes, whereas mAb PG9 was provided by Drs. Wayne Koff (International AIDS Vaccine Initiative) and Dennis Burton (Scripps Institute).

### Antigenicity testing of gp120/mAb complexes

Immune complexes of gp120 and anti-gp120 mAbs were made by mixing 2 μg/mL gp120_JRFL_ or gp120_A244_ with 4 μg/mL mAb in an Eppendorf LoBind Protein 96-well plate (Sigma) and incubating them at 37°C for 3 h. The complexes were then serially diluted 2-fold, added to ELISA plates, and incubated overnight at 4°C. Plates were washed and blocked with RPMI medium containing 15% FBS and 1% BSA for 1.5 h at 37°C. To assess changes in antigenicity of gp120 complexed with vs. without mAb, biotinylated mAbs against different gp120 epitopes (V2i, V2q, CD4bs, V3, C2) were added. Detection of biotinylated mAb binding was done with alkaline phosphatase-conjugated streptavidin, followed with alkaline phosphate substrate (Sigma) or PhosphaGLO AP substrate (VWR) as described above.

Antigenicity evaluation was also performed using Fortebio Octet BioLayer Interferometry. For this assay, a biotinylated anti-V3 mAb 694/98D (at a saturating concentration of 20 μg/mL) was applied onto streptavidin sensors, and the kinetics of gp120 interaction with the anti-V3 mAb were monitored over the designated time period.

### Immunization protocol

BALB/c mice (female, >6 weeks old, 4 to 5 animals per group) were injected subcutaneously with immune complexes or Env protein alone (3 μg gp120 or gp140 plus/minus 9 μg mAb per dose). Immunogens were mixed with 25 μg monophosphoryl lipid A (MPL; Sigma) and 250 μg dimethyldioctadecylammonium (DDA; Sigma) in 100 μl per dose. Animals were immunized 4 times at 2–3 weeks intervals. Blood was collected 2 weeks after the last immunization, and sera from each group were pooled. For gp120_JRFL_ and gp120_JRFL_/CD4bs 654 groups, additional animals were immunized, and sera were collected and tested individually. Animal studies were carried out according to the protocol approved by the Institutional Animal Care and Use Committee.

### ELISA to test serum Ab levels

Levels of Abs specific for gp120, V1V2, and V3 in immune sera were measured by ELISA as described previously (16, 17). Antigens were coated on ELISA plates and reacted with serially diluted mouse sera. Serum IgG binding was detected using alkaline-phosphate-conjugated secondary Abs. After the addition of p-nitrophenyl phosphate substrate (Sigma) or PhosphaGLO AP substrate (VWR), plates were read by a spectrophotometer at 405 nm or a luminometer, respectively. Data are reported as optical density 405 (OD_405_) or relative luminescence unit (RLU).

Competitive ELISA was performed according to a published protocol (37). Serially diluted sera were incubated for 10 min at room temperature with V1V2-1FD6 antigen precoated on ELISA plates (Immulon 4HBX; Thermo Scientific) at 1 μg/mL, and then incubated for 2 h with biotinylated V1V2 mAb (830A or PG9) at a concentration predetermined in competition with its nonbiotinylated counterpart. Plates were washed with PBS containing 0.02% Tween 20 and then treated for 1 hour at room temperature with streptavidin-horseradish peroxidase (Pierce) and read at 450 nm. A reduction in signal was calculated as % inhibition of biotinylated mAb binding to plate-bound antigen.

### HIV neutralization

Virus neutralization was measured using HIV-1 pseudoviruses with TZM-bl target cells as described previously (20). HIV-1 pseudoviruses were produced in transfected 293T cells using a ProFection kit (Promega) or polyethylenimine (PEI) MAX40,000 (Polysciences). Prior to testing in the neutralization assay, sera were heat-inactivated (56°C for 30 min). Virus was incubated for 1 h at 37°C with serially diluted sera and then added to TZM-bl cells in the presence of diethylaminoethyl-dextran (Sigma). Virus infection was determined after 48 h using the Bright-Glo Luciferase Assay System (Promega). For peptide absorption assay, V3 peptide (40 μg/mL) was added to serum 1 h before the addition of virus.

### V2-α4β7 blocking assay

This assay was performed as described (38). Streptavidin-coated plates were incubated with biotinylated cyclic V2 peptides (5 μg/mL) for 1 h at 37°C. Peptide-coated plates were incubated with dilute immune sera or control sera for 45 min at 37°C. After incubation, the plates were washed and α4β7^+^ RPMI8866 cells (2 × 10^5^/well) were added to the peptide-coated plates for 1 h at 37°C. For positive control, α4β7^+^ RPMI8866 cells were pre-incubated with anti-α4 mAb HP2/1. Cyclic V2 peptides of MN and 92TH023 were used to test sera of mice immunized with gp120_JRFL_ and gp120_A244_ immune complexes, respectively. After washing to remove nonadhering cells, remaining adhered cells were detected by AlamarBlue® dye (ThermoFisher). Plates were incubated at 37°C, 5% CO_2_ for 8 h, and fluorescence (excitation 570; emission 590) was measured at 2 h intervals. Data are presented as % inhibition (fluorescence from wells incubated with sera divided by fluorescence from wells incubated without sera and then subtracted by 100).

### ADCP

Measurement of ADCP was done as described (16), using human THP-1 cells and fluorescent NeutrAvidin beads (1-μm diameter; ThermoFisher Scientific) precoated with gp120 or V1V2-1FD6 antigens. Coated beads were incubated with serially diluted sera for 2 h at 37°C, washed, and added to THP-1 cells. After overnight incubation, phagocytosis was measured by flow cytometry. ADCP scores were calculated as: (% bead-positive cells × MFI of bead-positive cells)/10^6^, where MFI is mean fluorescence intensity. For comparison, ADCP activity was also evaluated with murine RAW264.7 cells.

### Statistical analysis

Statistical analyses were performed with t test or two-way ANOVA using GraphPad Prism 7.

## Results

### Alterations of V1V2 and V3 epitopes on gp120/mAb complexes vs. uncomplexed gp120

HIV-1 gp120 proteins, particularly their V1V2 and V3 regions, display a high degree of structural flexibility; this flexibility has been implicated in conformational masking of Ab epitopes in these regions (39–41). Our previous studies have shown that the interaction of gp120 with a CD4bs-specific mAb caused allosteric effects that stabilized the V3 loop for better Ab recognition and also rendered the gp120 protein more resistant to proteolytic degradation (18, 19, 25), although evidence for improved Ab recognition and preservation of V1V2 was lacking. To further investigate allosteric changes induced by various anti-gp120 mAbs and their effects on V1V2 and V3 epitopes, gp120 B.JRFL or gp120 AE.A244 in complex with different anti-gp120 mAbs were probed with a panel of mAbs specific for V1V2, V3, and other regions of gp120. Immune complexes were prepared by preincubating gp120 with each mAb in a molar ratio of 1:2.

**Figure 1:**
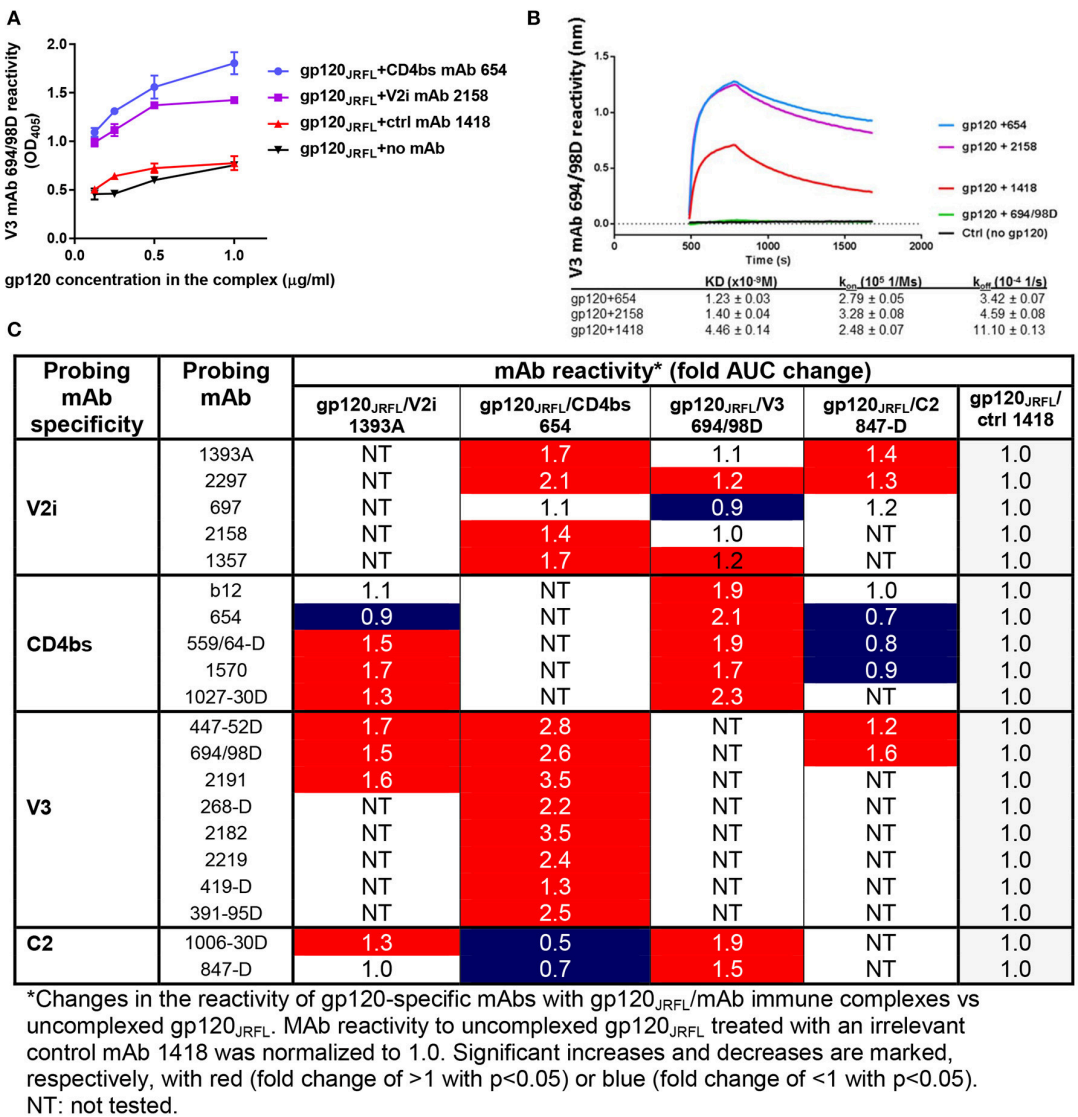
Changes in antigenicity of gp120_JRFL_ upon immune complex formation. **(A)** Immune complexes were prepared by incubating gp120_JRFL_ with different gp120-specific mAbs (molar ratio of 1:2). Serially diluted complexes were then coated onto ELISA plates and probed with biotinylated mAbs. Representative data are shown depicting the binding of biotinylated anti-V3 mAb 694/98D to gp120_JRFL_ in complex with CD4bs mAb 654 or V2 mAb 2158 as compared with gp120_JRFL_ treated with an irrelevant parvovirus-specific mAb 1418 or no mAb. **(B)** The immune complexes were also tested by BLI using Fortebio Octet for their relative reactivity with mAb 694/98D that was immobilized on the biosensor tip. **(C)** This panel summarizes ELISA data showing fold changes in mAb reactivity to different gp120_JRFL_/mAb complexes vs. uncomplexed gp120_JRFL_ (gp120_JRFL_ plus control mAb 1418). AUC: area under titration curve.

Figures 1A,B show improved binding of V3-specific mAb 694/98D, as measured by ELISA and Octet BioLayer Interferometry (BLI), when gp120_JRFL_ was complexed with CD4bs mAb 654 or V2i mAb 2158, in comparison with uncomplexed gp120_JRFL_ or gp120_JRFL_ treated with an irrelevant mAb against parvovirus (1418). We further probed the antigenicity of gp120_JRFL_ in complex with V2 mAb 1393A, CD4bs mAb 654, V3 mAb 694/98D, or C2 mAb 847-D with a larger panel of biotinylated mAbs against V2i, CD4bs, V3, and C2 in ELISA. Antigenicity changes were assessed by comparing the complexes with uncomplexed gp120_JRFL_ treated with irrelevant mAb 1418. The data in Figure 1C reveal significant allosteric alterations in each of the gp120/mAb complexes that resulted in increased or decreased gp120 recognition by particular mAbs. Thus, the gp120_JRFL_/1393A V2i mAb complex had greater reactivity with many, but not all, mAbs specific for V3, CD4bs, and C2. In contrast, the gp120_JRFL_/654 CD4bs mAb complex showed enhanced recognition by almost all V3 and V2i mAbs and reduced reactivity with C2 mAbs. gp120_JRFL_ in complex with the 694/98D V3 mAb had stronger reactivity with the CD4bs and C2 mAbs, but its reactivity with V2 mAbs was minimally changed. The gp120_JRFL_/847D C2 mAb complex displayed yet a different pattern, as it demonstrated reduced reactivity with many CD4bs mAbs and slightly better reactivity with V3 and V2 mAbs.

**Figure 2:**
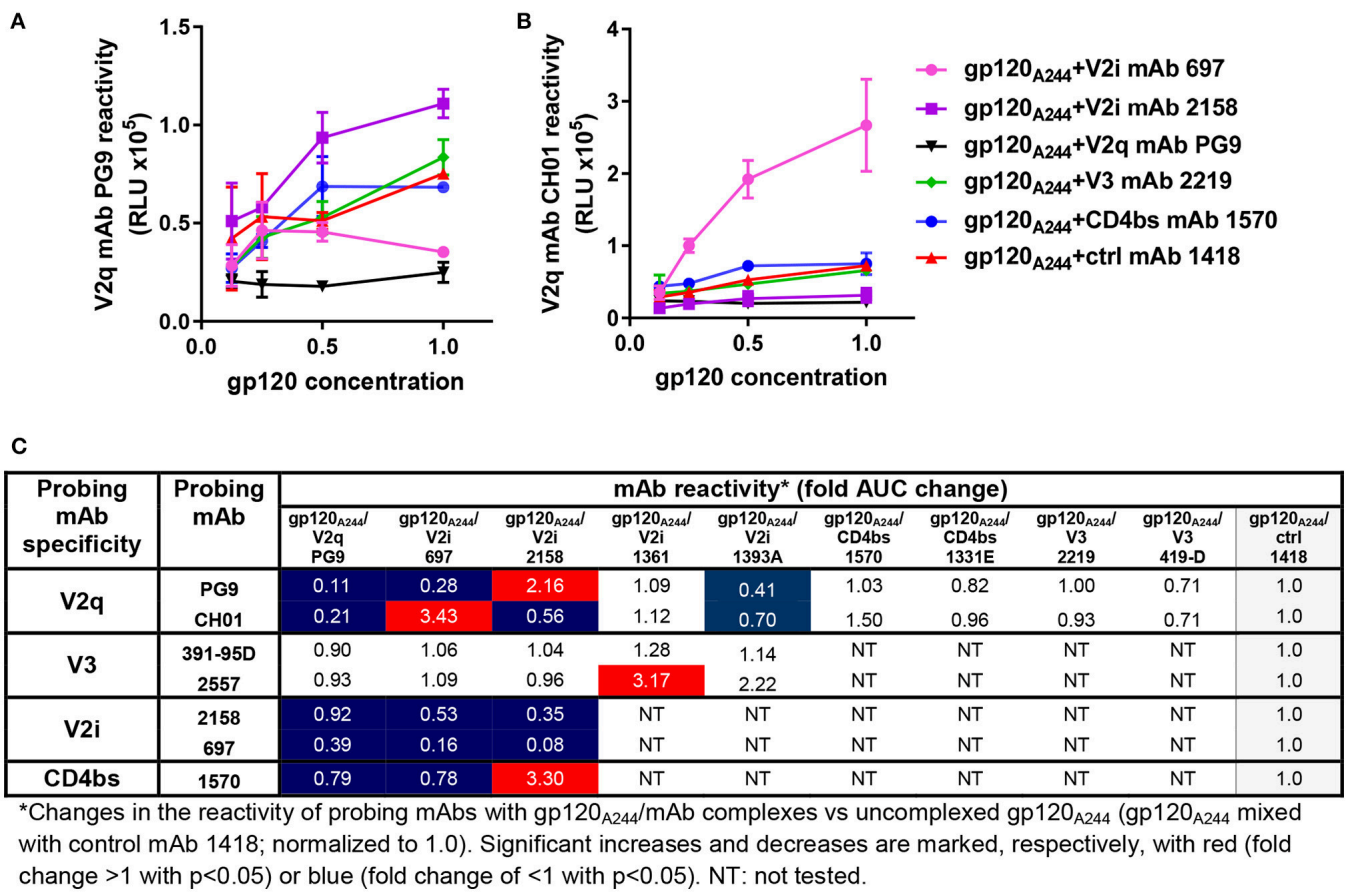
Changes in antigenicity of gp120_A244_ upon immune complex formation. Immune complexes made with gp120_A244_ and gp120-specific mAbs (molar ratio of 1:2) were tested in ELISA for reactivity with biotinylated mAb probes as described in Figure 1. **(A–B)** Representative data depicting altered reactivity of biotinylated anti-V2q mAbs PG9 **(A)** and CH01 **(B)** to gp120_A244_ complexed with V2i mAb 2158, V2i mAb 697, or V2q mAb PG9 relative to reactivity with uncomplexed gp120_JRFL_ mixed with control mAb 1418 are shown. **(C)** This panel summarizes fold changes in mAb reactivity to different gp120_A244_/mAb complexes vs. uncomplexed gp120_A244_ (gp120_A244_ + control mAb 1418). RLU: relative luminescence unit from ELISA with PhosphaGLO AP substrate. AUC: area under titration curve.

We also probed gp120_A244_/mAb complexes vs. gp120_A244_ with a panel of biotinylated mAbs that target the V1V2 domain (PG9, CH01, 2158, 697), the V3 loop (391-95D, 2557), and the CD4 binding site (1570) (Figure 2). Among the V1V2 mAb probes, PG9 and CH01 recognize quaternary glycan-bearing V2q epitopes on the trimeric V1V2 apex (42, 43), whereas 2158 and 697 bind to the V2i epitopes overlapping with or near the integrin α4β7-binding motif on the nonglycosylated underbelly of the V1V2 beta-barrel structure [(37, 44) and Kong et al. unpublished data]. Using mAbs recognizing these highly conformation-dependent epitopes, we found marked allosteric changes in the V1V2 domain that were induced upon the binding of gp120_A244_ by different mAbs. Notably, gp120_A244_ in complex with V2i mAb 2158 showed increased PG9 (V2q) reactivity and decreased CH01 (V2q) reactivity in comparison with uncomplexed gp120_A244_ treated with an irrelevant mAb 1418, whereas the gp120_A244_/697 V2i mAb complex displayed an opposite pattern of reactivity with PG9 and CH01 (Figures 2A,B). The data are in line with the crystallographic data showing the flexibility of V1V2 to adopt distinct structural configurations upon binding by different V1V2 mAbs, including V2q mAb PG9 (43) and V2i mAb 830A (45). gp120_A244_/2158 and gp120_A244_/697 complexes also had respectively increased and decreased 1570 (CD4bs) reactivity, but no change was seen in reactivity with V3 mAbs 391-95D or 2557 (Figure 2C). For control, the binding of biotinylated V2i mAbs 2158 and 697 was also assessed; poor or no reactivity was detected, affirming full occupancy and stable immune complex formation between gp120_A244_ with each of the V2i mAbs.

In contrast, the gp120_A244_/1361 V2i complex displayed no alteration in reactivity with V2q mAbs PG9 and CH01 but was recognized more strongly by one of the V3 mAbs (2557) (Figure 2C). Yet, the gp120_A244_/1393A V2i complex had another distinct pattern, showing reduced reactivity with both PG9 and CH01. We also tested gp120_A244_ in complex with V3 mAbs (2219, 419) or CD4bs mAbs (1570, 1331) and found that these complexes had unchanged reactivity with PG9 and CH01 vs. uncomplexed gp120_A244_ (Figure 2C). Altogether, the data presented here demonstrate that significant antigenic changes are induced in gp120 when gp120 forms immune complexes with mAbs; these changes may affect gp120 immunogenicity *in vivo*.

### Induction of Ab responses against V1V2 and V3 by immunization with gp120_JRFL_/mAb complexes formed with different mAbs

To assess whether immunization with immune complexes vs. uncomplexed gp120 resulted in altered Ab responses especially to V1V2 and V3, a set of experiments was conducted to test gp120 B.JRFL in complex with human IgG1 mAbs against C2 (1006-30D), V2i (2158), CD4bs (654), and V3 (1006-15D). Immune complexes were prepared by preincubating gp120_JRFL_ with each mAb in a molar ratio of 1:3, mixed with adjuvant MPL/DDA, and injected subcutaneously to BALB/c mice 4 times at 2- to 3-week intervals at a dosage of 3 μg gp120 and 9 μg mAb in 25 μg MPL and 250 μg DDA per injection per animal. Mice immunized with gp120 alone or PBS (no gp120) and adjuvant served as controls. Sera were collected 2 weeks after the last immunization and analyzed in ELISA for binding Ab levels.

**Figure 3:**
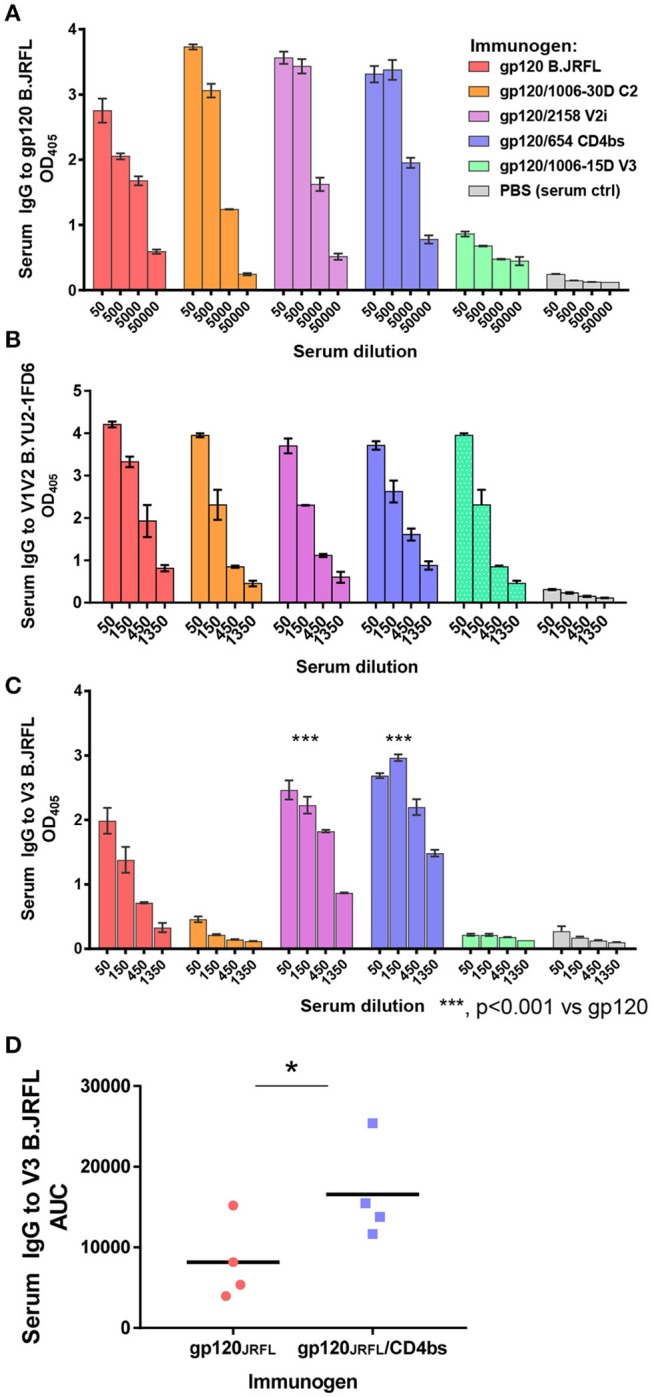
Serum Ab responses induced by vaccination with gp120_JRFL_/mAb complexes. BALB/c mice were immunized with gp120 B.JRFL complexed with human IgG1 mAbs of defined specificity—C2 (1006-30D), V2i (2158), CD4bs (654), V3 (1006-15D)—or with no mAb. Immune complexes were administered 4 times subcutaneously in the presence of adjuvant MPL/DDA. Mice immunized with PBS and adjuvant (no gp120) served as negative controls. **(A–C)** Pooled sera collected 2 weeks after the last immunization were tested in ELISA for IgG reactivity against gp120 **(A)**, V1V2 **(B)**, or V3 **(C)**. **(D)** Sera from individual mice immunized with gp120_JRFL_ vs. gp120_JRFL_/CD4bs mAb 654 were also tested for ELISA reactivity against V3. AUC: area under the titration curve of each serum sample; OD_405_: optical density at 405 nm obtained from designated serum dilution in ELISA with p-nitrophenyl phosphate substrate. ^*^p < 0.05.

ELISA data in Figure 3A show that the levels of serum IgG to gp120 B.JRFL were similarly high in mice immunized with gp120_JRFL_/1006-30D C2, gp120_JRFL_/2158 V2i, gp120_JRFL_/654 CD4bs, and uncomplexed gp120_JRFL_, whereas the gp120_JRFL_/1006-15D V3 group displayed a weak response. The levels of IgG binding to V1V2, as detected with a subtype B V1V2-YU2 on the 1FD6 scaffold, were equally high in all groups (Figure 3B). In contrast, V3-specific IgG levels varied greatly (Figure 3C). The gp120_JRFL_/1006-30D C2 and gp120_JRFL_/1006-15D V3 groups had a minimal anti-V3 Ab response, similar to the PBS control group, whereas the gp120/2158 V2i and gp120/654 CD4bs groups showed an enhanced anti-V3 Ab response, with half-max (ED_50_) titers of 600-1350 vs. 200 for gp120-immunized mice. Higher levels of V3-binding Abs were also evident in the sera of individual animals immunized with gp120_JRFL_/654 CD4bs vs. gp120_JRFL_ (Figure 3D). This increased anti-V3 Ab response corroborated previous findings with complexes made of gp120_LAI_/2158 and gp120_LAI_/654 (20), confirming the improved V3 immunogenicity of immune complexes made of gp120 and mAbs against V2i or the CD4bs.

The data in Figure 3 show that, unlike immune complexes made with mAbs to C2, V2i, and CD4bs, the gp120_JRFL_/V3 mAb complex induced a weak Ab response to gp120 and an undetectable Ab response to V3. Suppression of Ab response by the gp120_JRFL_/V3 mAb complex may be explained by mAb steric hindrance to the V3 crown, one of the most immunogenic sites on gp120. Indeed, inhibition of gp120- and V3-specific Ab induction was observed previously with an immune complex composed of gp120 B.LAI and V3 mAb 694/98D (17).

### Fine specificity of V3- and V1V2-specific Abs induced by gp120_JRFL_ vs. gp120_JRFL_/mAb complexes

**Figure 4:**
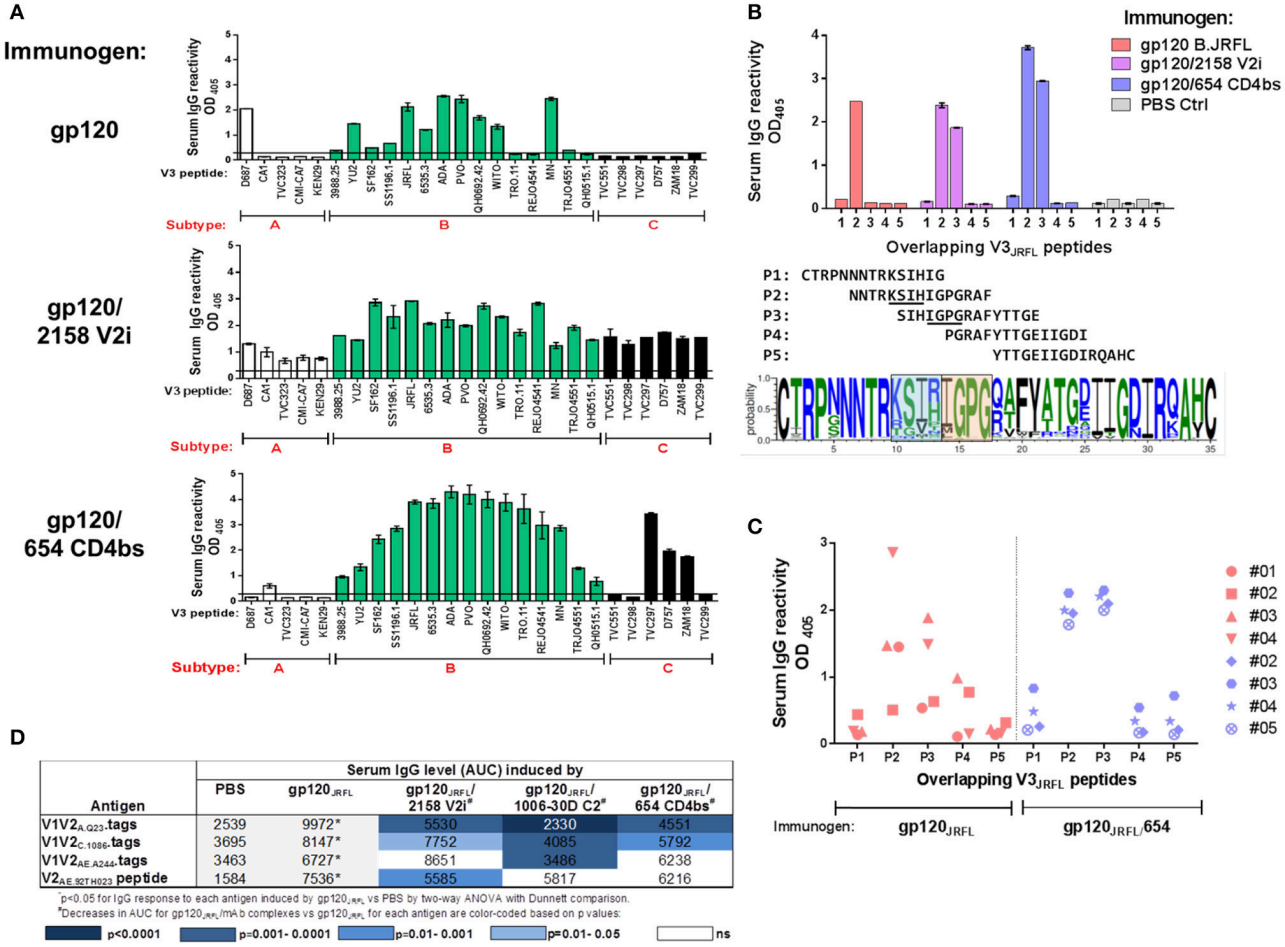
V3-specific Ab responses induced by gp120_JRFL_/mAb complexes vs. uncomplexed gp120_JRFL_**. (A)** Sera from mice immunized with gp120 B.JRFL in complex with V2i mAb 2158, CD4bs mAb 654, or no mAb were tested in ELISA for IgG reactivity against V3 peptides of HIV-1 subtypes A, B, and C. **(B)** Overlapping V3 peptides P1 to P5 were used for epitope mapping. Sequence logo depicting V3 amino acid variations is included to show the central V3 regions that are targeted by Abs induced by gp120_JRFL_ (blue-shaded box) vs. gp120_JRFL_/mAb complexes (orange-shaded box). **(C)** Sera from individual animals were tested for reactivity with overlapping V3 peptides P1 to P5 and demonstrated more uniform recognition of P2 and P3 peptides by all animals that received gp120_JRFL_/CD4bs mAb 654 vs. uncomplexed gp120_JRFL._. (**D**) Sera from mice immunized with gp120_JRFL_ or gp120_JRFL_/mAb complexes were also tested for IgG reactivity with different V1V2 antigens. AUC: area under the titration curve. PBS: Sera from control group that received PBS and adjuvant (no gp120).

To characterize V3-specific Ab responses generated in animals that received gp120_JRFL_ vs. gp120_JRFL_/V2i vs. gp120_JRFL_/CD4bs, Ab cross-reactivity was examined using a panel of 26 V3 peptides from HIV-1 subtypes A, B, and C (Figure 4A). Serum IgG of gp120-immunized mice reacted with 12 subtype B V3 peptides and one subtype A V3 peptide, although binding strength to some peptides was weak, being near the background level of control sera. Serum IgG of the gp120_JRFL_/V2i group, on the other hand, reacted broadly with all 26 peptides across three subtypes, although binding strength against subtype A peptides was lower relative to those against subtypes B and C peptides. IgG from mice immunized with the gp120_JRFL_/CD4bs mAb complex also was more cross-reactive than IgG from gp120-immunized animals but was not reactive with four subtype A peptides and three subtype C peptides. Nevertheless, binding strength against most subtype B peptides was superior compared with those attained by the other two groups. These data demonstrate significant improvement in the breadth of V3-specific Abs induced by gp120_JRFL_/V2i and gp120_JRFL_/CD4bs complexes compared with uncomplexed gp120_JRFL_.

Increased cross-reactivity of anti-V3 Ab responses in animals immunized with immune complexes vs. uncomplexed gp120 may indicate that distinct regions of V3 are targeted by these complexes. To examine this possibility, we evaluated serum IgG reactivity with five overlapping peptides that span the entire V3 loop from autologous B.JRFL. The results demonstrate that Ab response in gp120_JRFL_-immunized mice targeted solely peptide 2 (Figure 4B). In contrast, Abs induced in mice immunized with gp120_JRFL_/CD4bs mAb and gp120_JRFL_/V2i mAb complexes reacted with peptides 2 and 3. Uniform Ab reactivity to both peptides was also observed when sera from individual animals in the gp120_JRFL_/CD4bs mAb group were tested (Figure 4C). In contrast, no consistent pattern was seen among gp120_JRFL_-immunized animals.

A sequence logo illustrating amino-acid conservation and variability across the V3 loop among HIV-1 sequences in the Los Alamos National Laboratory database indicates that the middle portion of peptide 2 contains variable amino acids at positions 305 to 308, whereas the region common to peptides 2 and 3 is centered at the more conserved IGPG motif at the tip or arch of the V3 crown (Figure 4B). Altogether, the data in Figure 4 demonstrate a subtle change in fine specificities of V3-specific Ab responses induced by gp120_JRFL_/mAb complexes compared with uncomplexed gp120_JRFL_; this epitope shift influences the breadth of V3 Ab reactivity across diverse HIV-1 subtypes.

In contrast to the greater V3 Ab cross-reactivity we observed in Figure 4A, the V1V2 Ab response induced by gp120_JRFL_/mAb complexes was more subtype-restricted. Although responses to B.YU2 V1V2-1FD6 were similarly robust among groups immunized with gp120_JRFL_ and gp120_JRFL_/mAb complex (Figure 3B), responses to V1V2 and V2 antigens from subtypes A, C, and CRF_01.AE were mostly reduced in animals immunized with gp120_JRFL_/mAb complexes vs. uncomplexed gp120_JRFL_ (Figure 4D). Moreover, we noted distinct patterns of V1V2 cross-reactivity in the different groups: Ab response elicited by the gp120_JRFL_/C2 complex poorly recognized V1V2-tags of A.Q23, C.1086, and AE.244. The gp120_JRFL_/V2i complex generated Abs that reacted more weakly with V1V2 A.Q23, V1V2 C.1086, and cyclic V2 AE.92TH023 peptide. The gp120_JRFL_/CD4bs complex induced lower levels of Abs against tag-V1V2 of A.Q23 and C.1086. These data indicate that, although immunization with gp120/mAb complexes generated V3 Abs of higher titer and greater breadth, these complexes did not induce a greater level of V1V2 Abs, and V1V2 Abs that were elicited had reduced cross-reactivity.

### Induction of V3 and V1V2 Ab responses by gp120_A244_/mAb and gp140_CN54_/mAb complexes

**Figure 5:**
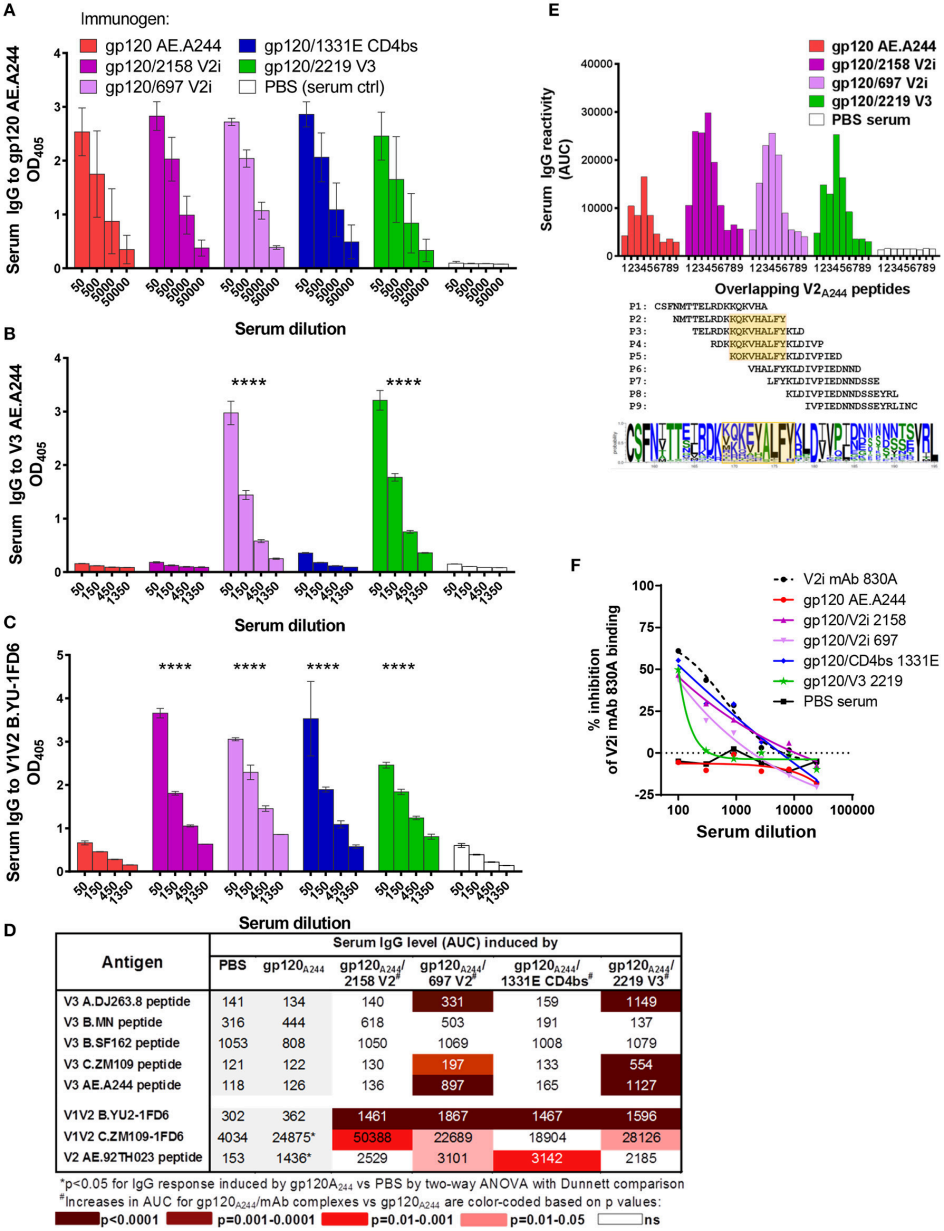
Serum Ab responses induced by gp120_A244_/mAb complex vaccines. Mice were immunized with gp120 AE.A244 in complex with human IgG1 mAbs specific for V2i (2158), V2i (697), CD4bs (1331E), V3 (2219), or with no mAb in the presence of adjuvant MPL/DDA. **(A–C)** Sera collected 2 weeks after the fourth immunization were tested in ELISA for IgG reactivity to gp120 **(A)**, V3 **(B)**, and V1V2 **(C)**. **(D)** Sera were also evaluated for cross-reactivity with V3 and V1V2 from viruses of different HIV-1 subtypes. **(E)** Further mapping of V2 epitope was performed using overlapping V2 peptides P1 to P9. V2 sequence logo is shown to indicate amino-acid variability within the defined epitope region (orange-shaded box). **(F)** Sera were subjected to competition ELISA using V1V2 C.ZM109-1FD6 to assess the presence of V1V2-specific serum Abs able to compete with conformation-dependent V2i mAb 830A. ^****^p < 0.0001.

A second set of experiments was performed to evaluate the immunogenicity of immune complex vaccines made with gp120 AE.A244. The complexes were formed with V2i mAbs 2158 and 697, CD4bs mAb 1331E, and V3 mAb 2219, each of which displayed binding affinity for gp120 AE.A244. Immunization with gp120_A244_/mAb complexes showed a different result from that of the gp120_JRFL_/mAb complex vaccines. Uncomplexed or complexed gp120_A244_ elicited similarly high levels of serum IgG against gp120 (Figure 5A). However, Ab response to V3 was induced only by gp120_A244_/697 V2i and gp120_A244_/2219 V3 complexes and not by gp120_A244_/2158 V2i, gp120_A244_/1331E CD4bs, or uncomplexed gp120_A244_ (Figure 5B). The V3-specific Ab response had limited breadth (Figure 5D): gp120_A244_/697 V2i induced V3 Abs reactive mainly to the autologous V3 AE.A244 and weakly recognizing the other V3 peptides tested; gp120_A244_/2219 V3 stimulated V3 Abs that were reactive with AE.A244, C.ZM109, and AG.DJ263.8 but failed to recognize B.MN and B.SF162. These data demonstrate that gp120_A244_/mAb complexes did not promote induction of broadly reactive V3 Abs.

Interestingly, each of the gp120_A244_/mAb complexes elicited a greater level of V1V2 Abs than its uncomplexed counterpart. Enhanced V1V2 Ab responses were detectable against heterologous B.YU2 V1V2 presented on the 1FD6 scaffold (Figure 5C) and, to a lesser extent, against C.ZM109 V1V2-1FD6 and cyclic V2 AE.92TH023 peptide (Figure 5D). Enhanced Ab responses were also observed against overlapping V2 AE.A244 peptides (Figure 5E). With these overlapping peptides, V2-specific responses induced by gp120_A244_/2158 V2i, gp120_A244_/697 V2i, gp120_A244_/2219 V3, and uncomplexed gp120_A244_ were mapped to the same region, which encompasses the C strand in the V1V2 domain (amino acid positions 169 to 177, based on HXB2 numbering) (Figure 5E). This region is relatively conserved at the last 4 positions (AELY); the remaining residues are highly variable among HIV-1 isolates. Using competition ELISA with conformation-dependent V2i mAb 830A, we further saw that sera from animals immunized with gp120_A244_/mAb complexes inhibited the binding of 830A more than did sera from gp120_A244_-immunized animals; in fact, the latter were indistinguishable from the PBS control group (Figure 5F). These results indicate the ability of gp120_A244_/mAb complexes to elicit greater Ab responses to V1V2, with some degree of cross-reactivity. Nonetheless, no competition was detected with V2q mAb PG9 (Figure S1), indicating that improved PG9 antigenicity displayed by the gp120_A244_/2158 V2i mAb complex was insufficient to induce PG9-like Ab responses *in vivo*.

Altogether, data in Figures 3–5 demonstrate that immunization with gp120/mAb complexes vs. uncomplexed gp120 augments the induction of Ab responses to V1V2 and V3, but the capacity to elicit more broadly reactive Abs varies depending on both Env strain and mAb used to form the complexes. These results are also congruent with findings from a third set of experiments which tested an immune complex vaccine made with gp140 C.CN54, a non-native uncleaved trimeric Env from a subtype C clone p97CN54 used in a phase I clinical vaccine trial in the UK (46, 47). Mice immunized with gp140 C.CN54 complexed with the CD4bs mAb 654 showed a pattern of Ab response modulation: as compared with uncomplexed gp140_CN54_, the gp140_CN54_/654 complex generated a higher Ab response to the homologous V3_CN54_ and higher or similar Ab responses to V1V2_A.Q23_ and other V1V2 (Figure S2).

### Functional activity of Abs elicited by different gp120/mAb complexes

To test the antiviral potential of Abs induced by immune-complex vaccines, sera from animals immunized with gp120_JRFL_/mAb and gp120_A244_/mAb complexes were evaluated for the capacity to (i) neutralize HIV pseudoviruses in the standard TZM.bl assay, (ii) block Env interaction with the integrin α4β7, and (iii) mediate ADCP with THP-1 effector cells.

**Figure 6:**
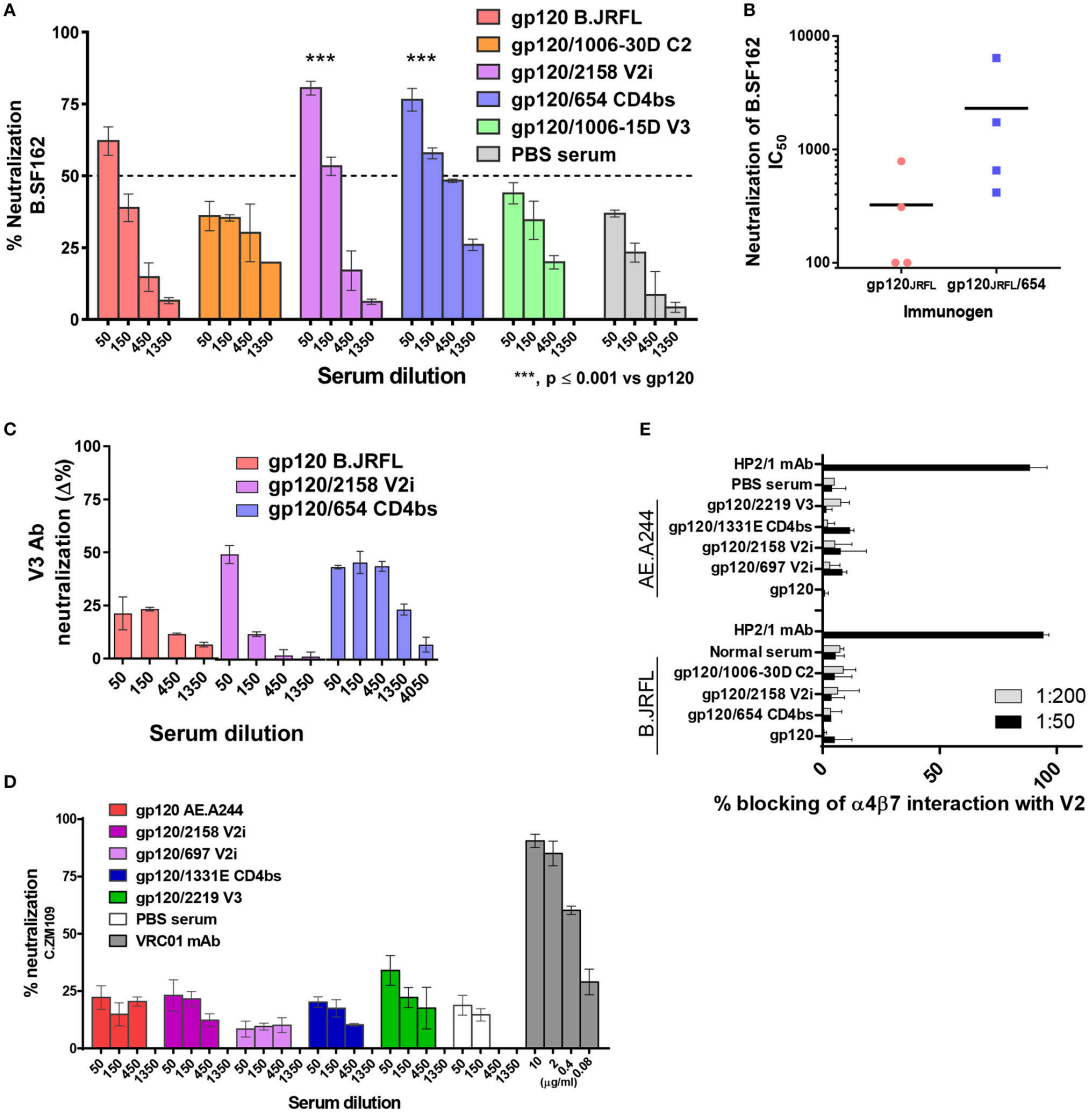
Inhibitory activity of immune sera from animals that received gp120/mAb complexes vs. uncomplexed gp120. **(A)** Pooled sera from mice immunized with gp120_JRFL_/mAb complexes or uncomplexed gp120_JRFL_ were evaluated for neutralizing activity against HIV-1 B.SF162 using TZM.bl target cells. **(B)** Neutralization activity of individual animal serum from groups that received gp120_JRFL_ vs. gp120_JRFL_/CD4bs mAb 654 was also compared. **(C)** Neutralization mediated by V3-specific Abs was assessed by measuring neutralization activity of sera pretreated with or without V3 peptide (40 μg/ml). Δ% neutralization was calculated by subtracting % neutralization of untreated sera with that of V3 peptide-treated sera. **(D)** Likewise, sera from animals immunized with gp120_A244_/mAb complex or uncomplexed gp120_A244_ were tested for neutralizing activity against HIV-1 C.ZM109 using TZM.bl cells. CD4bs-specific mAb VRC01 was included as a positive control. **(E)** Sera from all groups of immunized mice were also tested for the capacity to block V2 interaction with the integrin α4β7. The α4-specific mAb HP2/1 served as a positive control. PBS: sera from control group that received PBS and adjuvant (no gp120).

Sera from mice immunized with gp120_JRFL_/mAb complexes and uncomplexed gp120_JRFL_ were tested for neutralizing activity against HIV-1 B.SF162, a tier 1a virus highly sensitive to V3 Abs (48). Higher neutralizing activity was detected in sera from the gp120_JRFL_/V2i 2158 and gp120_JRFL_/CD4bs 654 groups compared with the gp120_JRFL_-immunized group (IC_50_: 150-450 vs. 60), whereas <50% neutralization was achieved with sera from the gp120_JRFL_/1006-30D C2 and gp120_JRFL_/1006-18D V3 groups (Figure 6A), in correlation with V3-binding Ab levels (Figure 3C) (*p* = 0.017, *r* = 0.941 by Spearman test). Increased neutralization was also seen when individual sera from the gp120_JRFL_/654 CD4bs group were tested in comparison with those from the gp120_JRFL_ group (*p* = 0.038 by t test, Figure 6B). However, no neutralization was detected against autologous JRFL or other tier 2 isolates [data not shown and (20)]. To more directly assess the contribution of V3 Abs to SF162 neutralization, we pretreated sera with V3 peptide and measured reduction of neutralization. The data show that neutralization in sera from animals immunized with gp120_JRFL_, gp120_JRFL_/2158 V2, and gp120_JRFL_/654 CD4bs was mediated in part by V3-specific Abs, with the strongest V3 Ab neutralization seen in the gp120_JRFL_/654 CD4bs group (Figure 6C), indicating that the gp120_JRFL_/654 CD4bs immune complex was the most efficient immunogen for eliciting a neutralizing anti-V3 Ab response.

Sera from animals immunized with gp120_A244_ vs. gp120_A244_/mAb complexes were also tested for neutralizing activity. In contrast to gp120_JRFL_/mAb complexes (Figures 6A–C), none of the gp120_A244_/mAb complexes stimulated neutralizing Abs against tier 1b (C.ZM109) (Figure 6D) or tier 2 viruses (B.JRFL, C.CAP45) (data not shown). Similarly, mice immunized with uncomplexed gp120_A244_ had no detectable neutralizing Abs against these viruses.

We also measured the capacity of Abs from mice immunized with gp120_JRFL_/mAb complexes vs. uncomplexed gp120_JRFL_ to inhibit HIV Env binding to α4β7, an integrin that has been shown to interact with V2 to facilitate virus-cell interaction and virus transmission from cell to cell (38, 49). No inhibition was detected with sera from any group, similar to the lack of inhibition seen with normal sera (Figure 6E). Likewise, sera from mice immunized with gp120_A244_/mAb complexes or gp120_A244_ alone failed to inhibit V2 binding to α4β7.

**Figure 7:**
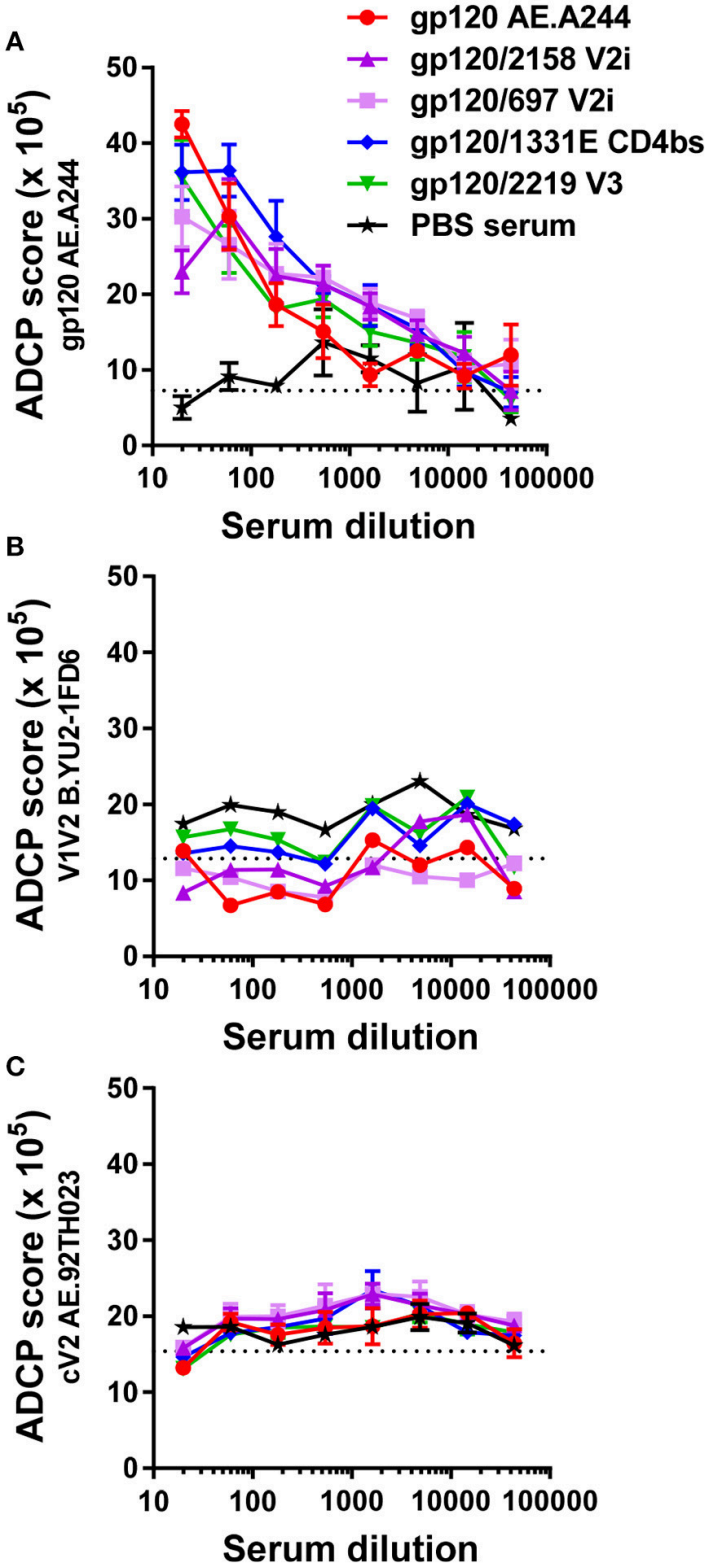
ADCP activity of immune sera from animals that received with gp120_A244_ or gp120_A244_/mAb complexes. **(A–C)** Pooled sera from groups of mice immunized with gp120 AE.A244 in complex with different mAbs or no mAb were tested for the ability to mediate ADCP using phagocytic THP-1 cells and fluorescent beads coated with gp120 **(A)**, V1V2-1FD6 **(B)**, or cyclic V2 peptide **(C)**. No significant difference was observed among the groups. Sera from animals that received adjuvant MPL/DDA and PBS (no gp120) were included as negative controls. ADCP score was calculated by multiplying the percentage of bead-bearing cells with the geometric mean intensity of the cells and subtracting the background score. Cutoff value (dotted lines) was determined based on ADCP scores of cells and beads without serum.

We subsequently asked whether Abs induced by gp120_A244_ had the capacity to mediate ADCP activity and whether induction of such Abs was augmented by immunization with gp120_A244_/mAb complexes. We tested ADCP against gp120_A244_, V1V2-1FD6, and cyclic V2 peptide (Figure 7), because enhanced V1V2-binding Ab responses were detected (Figure 5). Antigens coated on beads were treated with sera pooled from each group. Treated beads were incubated with THP-1 cells, and ADCP was measured. THP-1 is a human monocytic cell line with human FcɤRI and FcɤRII receptors (50, 51); however, the different mouse IgG subclasses can engage these Fcɤ receptors (52, 53), and as shown in Figure S3, ADCP activity of sera from gp120-immunized mice was similarly observed whether human THP-1 cells or mouse RAW264.7 cells were used. The data from ADCP assay using THP-1 cells showed that sera from animals immunized with gp120_A244_ and gp120_A244_/mAb complexes exhibited comparable levels of ADCP activity against gp120_A244_ (Figure 7A). However, no ADCP activity was detected against B.YU2 V1V2-1FD6 (Figure 7B) or AE.92TH023 V2 peptide (Figure 7C). Hence, although greater levels of V1V2-binding IgG were elicited by gp120_A244_/mAb complexes vs. gp120_A244_ (Figure 5), these V1V2-specific Abs did not have ADCP activity. Moreover, gp120_A244_-specific Abs with ADCP activity were induced, but they did not target V1V2 and the ADCP levels were not augmented by gp120_A244_/mAb complexes.

## Discussion

**Figure 8:**
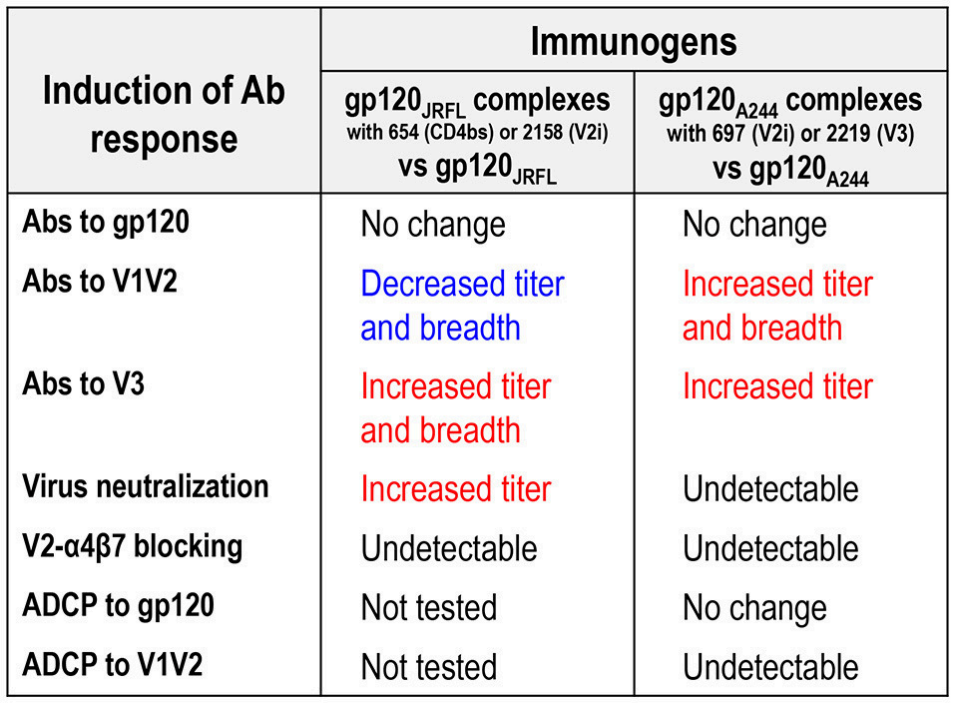
Differential modulation of Ab responses following immunization with gp120_JRFL_/mAb complexes vs. gp120_A244_/mAb complexes.

This study demonstrates the allosteric effects of mAbs that, upon binding to the HIV Env gp120 proteins, modulate the antigenicity and immunogenicity of the V1V2 and V3 regions of gp120. Hence, our data show that the gp120/mAb complexes, when utilized as vaccines, do not enhance overall Ab responses to HIV Env; rather, they influence the fine specificity of Ab responses, especially against the V1V2 and V3 regions. Notably, the gp120/mAb complexes that elicit higher titers of broadly reactive anti-V3 Abs do not exert the same effects on Ab responses to V1V2, and vice versa. As summarized in Figure 8, certain gp120_JRFL_/mAb complexes enhanced Ab responses to V3 and increased neutralization mediated by V3 Abs, consistent with our earlier studies with gp120_LAI_/mAb complexes (19, 20). This activity was determined by the specificity of mAbs used: only the complexes made of CD4bs mAb (654) and V2i mAb (2158) enhanced V3-specific Ab induction, while the complexes made of C2 mAb (1006-30D) and V3 mAb (1006-15D) caused suppression. Of note, vaccination with immune complexes bearing CD4bs mAb (654) and V2i mAb (2158) were associated with Ab responses that uniformly centered on the conserved GPG region at the tip of V3, which, correspondingly, broadened Ab reactivity to different HIV-1 subtypes. The ability to skew Ab response toward V3 was also seen previously with gp120 B.LAI in complex with another CD4bs mAb (559/64D) (17). In contrast, polyclonal V1V2 Ab responses were not enhanced upon immunization with the gp120_JRFL_/mAb complexes vs. uncomplexed gp120_JRFL_ and, in fact, demonstrated reduced titer and cross-reactivity.

The antigenic and immunogenic alterations observed with gp120_JRFL_/mAb complexes were not recapitulated by complexes made of gp120_A244_ (Figure 8). Indeed, an opposite pattern was observed with gp120_A244_/mAb complexes: the gp120_A244_/697 V2i and gp120_A244_/2219 V3 complexes elicited greater polyclonal Ab responses to V1V2 that displayed some degree of cross-reactivity as compared with uncomplexed gp120_A244_. Enhanced V3 Ab responses were also induced by these two complexes, but they had restricted breadth, recognizing V3 of subtypes A and C but not subtype B; this is most likely due to failure to recognize the GPGR motif characteristic of subtype B V3 (54).

Altogether, these data point to an important biological role of anti-Env Abs in determining Env immunogenicity. The data also suggest a potential use of Abs in combination with selected Env immunogens as vaccines to direct Ab responses toward or away from Env regions of interest. In support of this notion, past studies have showed that immunization with antigen-mAb complexes using HIV-1 gp120 core or the envelope antigen of tick-borne encephalitis virus resulted in modulation of the fine specificity of polyclonal Ab responses generated against the respective antigens, and that this activity was associated with epitope shielding or Ab-induced structural changes (55, 56). Our results are also congruent with earlier findings showing the contribution of passively transferred mAbs to accelerated emergence of neutralizing Abs against HIV (57) and enhanced cytotoxic T cell response to tumor antigens (58). Similarly, passive administration of the CD4bs-specific mAb b12 to newborn macaques prior to virus challenge augmented de novo Ab response to challenge SHIV virus in terms of gp120-binding and virus-neutralizing titers (59), corroborating the idea that preexisting or exogenously administered Abs influence the quality of immune responses to subsequent antigen exposure from infection or vaccination.

The results of the RV144 trial pointed us toward the protective potential of V1V2 Abs (3–7); therefore, we examined the antiviral capacity of V1V2 Abs induced by gp120_A244_/mAb complexes. In our study, polyclonal serum Abs against V1V2 of subtypes B, C, and CRF01.AE were induced to greater titers by these complexes, compared with uncomplexed gp120_A244_. Unlike uncomplexed gp120_A244_, gp120_A244_/697 V2i mAb and gp120_A244_/2219 V3 mAb also elicited polyclonal Ab responses to V3 that cross-reacted with V3 of C.ZM109 and A.DJ263.8. Nonetheless, no serum neutralizing activity was detected against C.ZM109, a tier 1b virus relatively sensitive to neutralization by V2i and V3 mAbs. Neutralization was similarly not observed against other viruses tested (data not shown). The elicited anti-V1V2 serum Abs also failed to inhibit Env binding to the integrin α4β7, which has been reported to be mediated in part by V1V2 and may promote HIV targeting of highly susceptible α4β7^+^ CD4 T cells (38, 49). Furthermore, we did not detect ADCP activity against V1V2 in the sera of animals immunized with gp120_A244_/mAb complexes. The ADCP assay was performed with a THP-1 cell line expressing human FcɤRI (CD64) and FcɤRII (CD32) receptors (51). These human Fc receptors are distinct from their murine counterparts; nonetheless, they have affinity for mouse IgG Fc (52) and vice versa (60). Indeed, gp120_A244_-specific ADCP activity from immunized sera was measurable using human THP-1 or murine RAW264.7 cells. It is unclear why V1V2 Abs from gp120_A244_/mAb-complex immunized animals lacked ADCP activity. Human mAbs specific for different V1V2 epitopes (V2i, V2p, and V2q) have been shown to display ADCP and ADCC activities (61, 62), and our data indicate that Abs that recognized V2 peptide (V2p-like Abs) and competed with V2i mAb 830A (V2i-like Abs) were present in the sera of animals immunized with gp120_A244_/mAb complexes. Nonetheless, fine differences in epitope specificity cannot be ascertained from this study. More specifically, as Fc functions are dictated by the efficiency with which Abs engage Fc receptors on effector cells, Fc-Fc receptor interactions may be influenced by the angle or direction by which Abs approach their targeted epitopes; such information can be gleaned only from crystallographic structures of V1V2/Ab complexes made of V1V2-specific Abs with and without ADCP functions.

Changes in gp120 immunogenicity that result from immune-complex formation are accompanied by significant antigenic and allosteric changes induced by gp120-mAb interaction. However, V1V2 and V3 antigenicity changes detected *in vitro* by mAb probes do not completely correlate with or explain the altered immunogenicity of these Env regions in the context of gp120/mAb complex vaccines. For example, gp120_JRFL_/654 CD4bs mAb complex displayed enhanced antigenicity of both V1V2 and V3, but immunization with this complex resulted only in enhanced V3 Ab response, while Ab response to V1V2 was reduced or unaltered. Similarly, gp120_A244_/2158 V2i mAb showed increased reactivity with V2q mAb PG9, but immunization with this complex did not induce PG9-like or PG9-competing Abs. Rather, induction of other V1V2-specific Abs (V2p- and V2i-like Abs) was augmented, although their antiviral functions remain unknown. The allosteric changes observed *in vitro* with the different gp120/mAb complexes were dependent on the mAb specificity dictated by the Fab portion of the mAb. Alterations in V1V2 and V3 immunogenicity detected *in vivo*, on the other hand, may also be due to Fc contribution. Indeed, immune complexes have been shown to be more potent inducers of Abs against hepatitis B virus (63) and infectious bursal disease virus (64, 65), mainly due to their Fc functions. Engagement of inhibitory FcɤIIb receptors by immune complexes in rhesus macaques that received SIVmac239Δnef vaccination also has been suggested to dampen an innate immune response in vaginal mucosa and thus reduce virus acquisition upon pathogenic SIV challenge (66). Nonetheless, evidence from our earlier study showed that an increased V3 Ab response induced by vaccination with the gp120/654 CD4bs mAb complex was attainable in the absence of Fc: Similarly high levels of Ab response to V3 were generated following immunization with gp120 B.LAI in complex with Fab_2_ fragment or intact IgG of CD4bs mAb 654 (20). Comparable V3 Ab levels were also elicited by gp120 complexed with mAb 654 bearing human or murine Fc (unpublished data). Fc activity of immune-complex vaccines against other antigens such as hepatitis B virus surface antigen and Mycobacterium tuberculosis Ag85B (Pepponi et al.) has been attributed to improved antigen processing and presentation by antigen-presenting cells (APCs) that take up the immune complexes via Fc receptors (67, 68). However, the anti-CD4bs mAb 654 has been shown to obstruct gp120 proteolytic processing by APCs and prevent gp120 antigen presentation to CD4 T cells, due to high-affinity binding and retention of stable gp120/654 complex in the acidic endolysosomal environment of the APCs (24, 25). Immune complexes made with other anti-Env mAbs have not been investigated; these complexes may not exhibit the same properties and may require both Fab and Fc activities to augment the elicitation of Env-specific Ab response.

In conclusion, this study demonstrates the capacity of immune-complex vaccines composed of recombinant HIV Env proteins and anti-Env mAbs to modulate induction of Abs against immunogenic epitopes in V1V2 and V3. However, differential Ab responses are induced upon vaccination with immune complexes made with mAbs of distinct specificity and with Env proteins of gp120 B.JRFL, gp120 AE.A244, and gp140 C.CN54. Improving the breadth and potency of Ab responses to both V1V2 and V3 may require vaccination with a combination of multiple mAb-Env pairs with complementary immunogenic properties. These immune-complex vaccines would offer a practical new strategy for the development of an efficacious and safe prophylactic HIV vaccine.

## Author contributions

CH conceived the study, obtained funding, analyzed and interpreted data, and wrote the manuscript. RK designed and conducted immunization experiments, collected specimens, performed ELISA and neutralization assays, and analyzed data. CU and AF performed ADCP experiments. MJ analyzed data, performed statistical analyses, and prepared figures and tables. VI produced, purified, and conducted quality control of mAbs. KP and MR performed Env-α4β7 blocking experiments. LL and NL performed antigenicity testing of Env/mAb complexes. MT prepared reagents and assisted with immunization experiments. XJ and X-PK performed competitive ELISA experiments. SZ-P provided mAbs and funding.

### Conflict of interest statement

The authors declare that the research was conducted in the absence of any commercial or financial relationships that could be construed as a potential conflict of interest.
