# Supplementary figures and images for: Modulation of Antibody Responses to the V1V2 and V3 Regions of HIV-1 Envelope by Immune Complex Vaccines
Source: Front Immunol. 2018 Oct 26;9:2441. doi: 10.3389/fimmu.2018.02441 (PMC6212562; doi:10.3389/fimmu.2018.02441)

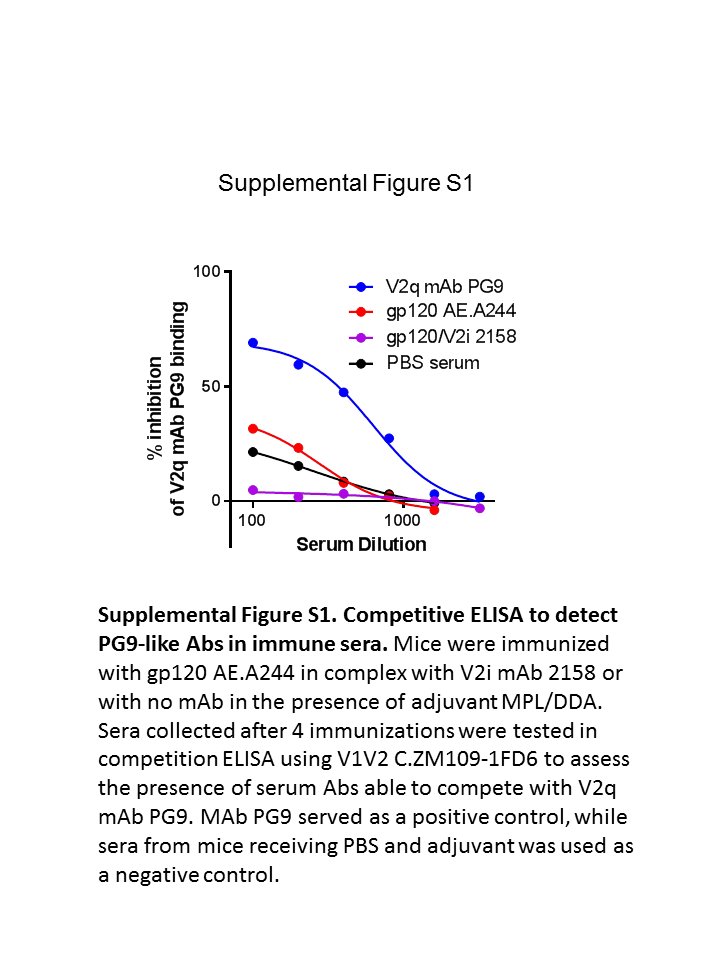

Supplement: Supplementary file 1 [file Image_1.TIF]

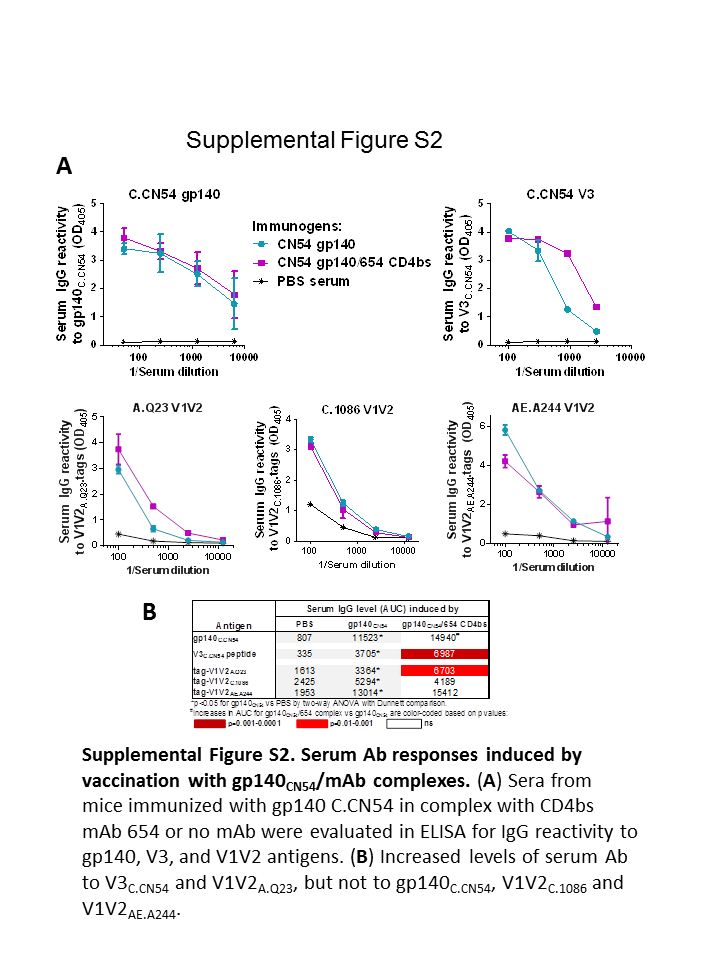

Supplement: Supplementary file 2 [file Image_2.TIF]

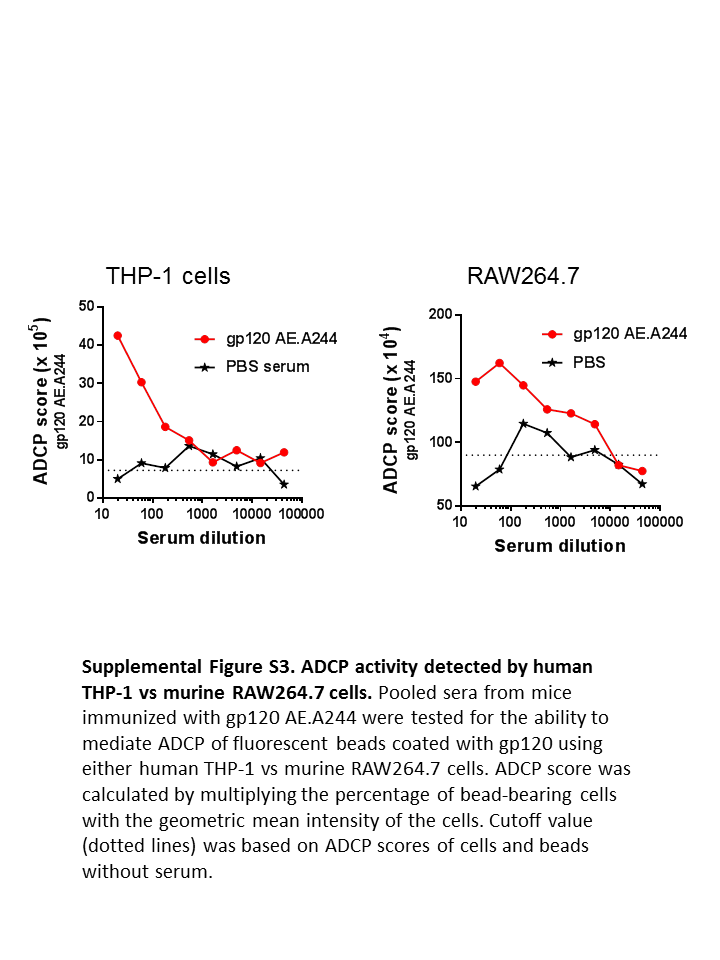

Supplement: Supplementary file 3 [file Image_3.TIF]
